# Supplementary material for: Reciprocity of thermal diffusion in time-modulated systems
Source: Nat Commun. 2022 Jan 10;13:167. doi: 10.1038/s41467-021-27903-3 (PMC8748696; doi:10.1038/s41467-021-27903-3)
Supplement: Supplementary file 1 — Supplementary Information [file 41467_2021_27903_MOESM1_ESM.pdf]

Supplementary Information

Reciprocity of thermal diffusion in time-modulated systems

Li et al.

This PDF file includes:

Supplementary Notes 1 to 5

Supplementary References

Supplementary Figures 1 to 6

Supplementary Note 1: Derivation of Eq. (2)

The first law of thermodynamics[0] requires that for a control volume V

$$E = Q_{\mathrm{cond}} + Q_{\mathrm{conv}} + Q_{\mathrm{inter}} + W \tag{S1}$$

where the rate of energy accumulation E is

$$E = \int_V \frac{\partial(\rho c T)}{\partial t} dV \tag{S2}$$

the net heat transfer by conduction Q_{cond} is

$$Q_{\mathrm{cond}} = -\int_A \mathbf{q}\cdot\mathbf{n}dA = -\int_V \nabla\cdot\mathbf{q}dV = \int_V \nabla\cdot(\kappa\nabla T)dV \tag{S3}$$

the net transfer of energy by fluid flow Q_{conv} is

$$Q_{\mathrm{conv}} = -\int_A \rho cT\mathbf{v}\cdot\mathbf{n}dA = -\int_V \nabla\cdot(\rho cT\mathbf{v})dV \tag{S4}$$

and we assume that there is no rate of internal heat generation ($Q_{\mathrm{inter}} = 0$) or net work transfer from the control volume to its environment ($W = 0$). Assembling expressions Eq.(S2)-(S4) into Eq.(S1), we obtain

$$\frac{\partial(\rho cT)}{\partial t} + \nabla\cdot(\rho cT\mathbf{v}) = \nabla\cdot(\kappa\nabla T) \tag{S5}$$

which can be reformulated as

$$\rho c\frac{\partial T}{\partial t} + \rho c\mathbf{v}\nabla T + cT\left[\frac{\partial\rho}{\partial t} + \nabla\cdot(\rho\mathbf{v})\right] = \nabla\cdot(\kappa\nabla T) \tag{S6}$$

Considering the conservation of mass, we have the continuity equation

$$\frac{\partial\rho}{\partial t} + \nabla\cdot(\rho\mathbf{v}) = 0 \tag{S7}$$

Then Eq.(S6) becomes

$$\rho c\frac{\partial T}{\partial t} + \rho c\mathbf{v}\nabla T = \nabla\cdot(\kappa\nabla T) \tag{S8}$$

Hence, Eq.(2) in the main text is obtained, which can also be written in the form as

$$\rho c\frac{DT}{Dt} = \nabla\cdot(\kappa\nabla T) \tag{S9}$$

where D/Dt represents the "material derivative" operator

$$\frac{D}{Dt} = \frac{\partial}{\partial t} + v_j\frac{\partial}{\partial x_j} \tag{S10}$$

Supplementary Note 2: Analytical model and solution of Eq. (5)

1D analytical model

A mass-conserving diffusive 1D heat transfer system under time modulation is governed by the convection-diffusion equation

$$\rho(x,t)c\frac{\partial T}{\partial t}+\rho(x,t)cv\frac{\partial T}{\partial x}=\frac{\partial}{\partial x}\left[\kappa(x,t)\frac{\partial T}{\partial x}\right] \tag{S11}$$

where the density ρ and thermal conductivity κ of the material are d-periodic functions of $\chi = x - v_0t$. Based on the periodicity of $\rho(\chi)$ and $\kappa(\chi)$, we write their expanded Fourier series as

$$\rho(\chi)=\sum_l \rho_l e^{il\beta\chi} \tag{S12}$$

$$\kappa(\chi)=\sum_l \kappa_l e^{il\beta\chi} \tag{S13}$$

where the integer index l takes 0, ±1, ±2, … and $\beta = 2\pi/d$. The coefficients satisfy $\rho_l = \rho_{-l}^*$ and $\kappa_l = \kappa_{-l}^*$ for the reality of the parameters, where the superscript star means complex conjugation.

By substituting $\rho(\chi)$ into the continuity equation $\partial\rho/\partial t + \partial(\rho v)/\partial x = 0$, we obtain

$$\frac{\partial(\rho v)}{x}=ilv_0\sum_l \rho_l e^{il\beta\chi} \tag{S14}$$

$$\rho v=v_0\sum_l \rho_l e^{il\beta\chi}+g(t)=\rho v_0+g(t) \tag{S15}$$

where $g(t)$ is d/v_0-periodic in order to reach stable state. Without loss of generality, we neglect this additional time dependence and simply let $g(t) = -\rho_0 v_0 + C$, which does not affect any of the conclusions. Then

$$\rho v=(\rho-\rho_0)v_0+C \tag{S16}$$

If we require that there is no accumulated net mass flux through the system, the average of ρv over a period of time should be 0, which gives $C = 0$. Otherwise, $C \neq 0$ means there exists a directional mass flux through the system.

Substituting Eq. (S16) into Eq. (S11) gives the general 1D convection-diffusion equation

$$\rho(\chi)c\frac{\partial T}{\partial t}+\left\{\left[\rho(\chi)-\rho_0\right]v_0+C\right\}c\frac{\partial T}{\partial x}=\frac{\partial}{\partial x}\left[\kappa(\chi)\frac{\partial T}{\partial x}\right] \tag{S17}$$

When $C = 0$, it is the case we discussed in the main text. When $C \neq 0$, there is additional constant mass flux C passing through the system, which could induce non-reciprocity.

Analytical solution of the general 1D convection-diffusion equation

After a variable change (x,t) to $(\chi = x - v_0 t,\ \tau = t)$, it is easy to see that Eq. (S17) is periodic on χ, so Floquet-Bloch theorem applies and gives a solution

$$T(\chi,\tau) = e^{iK\chi} e^{-i\Omega\tau} f(\chi) = e^{iKx} e^{-i(\Omega + k v_0)t} f(\chi) \tag{S18}$$

where Ω is the frequency and K is the Bloch wavenumber. Eq. (S18) describes a temperature profile characterized by Ω and K, and modulated by a d-periodic function $f(\chi)$. For time-harmonic solutions, the temperature profile should not vary with time, so $\Omega + Kv_0 = 0$. Then the solution at harmonic steady state becomes

$$T(x,t) = e^{iKx} f(\chi) = e^{\alpha x} f(\chi) \tag{S19}$$

where we rewrite the Bloch wavenumber as $K = -\alpha i$ for the convenience of following calculations. $f(\chi)$ is a d-periodic function that can be expanded as

$$f(\chi) = \sum_m F_m e^{im\beta\chi} \tag{S20}$$

where the integer index m takes 0, ±1, ±2, … and $F_0 = 1$.

Substituting Eq. (S19) into Eq. (S17), we have

$$\kappa f'' + \left[2\alpha\kappa + \kappa' + (\rho_0 v_0 - C)c\right] f' + \left[\alpha\kappa + \kappa' + (-\rho v_0 + \rho_0 v_0 - C)c\right]\alpha f = 0 \tag{S21}$$

Combined with Eqs. (S12), (S13) and (S20), we further deal with this simplified equation by Fourier series expansion, and write the nth order of Eq. (S21) as

$$\sum_m \left[(\alpha + i\beta n)(\alpha + i\beta m)\kappa_{n-m} - \alpha\rho_{n-m} c v_0 + \delta_{n,m}(\alpha + i\beta n)(\rho_0 v_0 - C)c\right] F_m = 0 \tag{S22}$$

where n =0, ±1, ±2, … and δ_{nm} is the Kronecker delta. For $n \neq 0$ and $m \neq 0$, F_m can be solved as

$$F_m = -\sum_{n\neq 0} A_{m,n}\alpha\left[(\alpha + i\beta n)\kappa_n - \rho_n c v_0\right] F_0 \tag{S23}$$

where $A_{m,n}$ is the m-row, n-column element of a matrix $[A_{m,n}]$, which is defined as

$$\left[A_{m,n}\right] = \left[(\alpha + i\beta n)(\alpha + i\beta m)\kappa_{n-m} - \alpha\rho_{n-m} c v_0 + \delta_{n,m}(\alpha + i\beta n)(\rho_0 v_0 - C)c\right]^{-1} \tag{S24}$$

Substituting Eq. (S23) into the 0th order of Eq. (S22) (n = 0) gives

$$\alpha^2 \sum_{m\neq 0}\left[(\alpha + i\beta m)\kappa_{-m} - \rho_{-m} c v_0\right] \sum_{n\neq 0} A_{m,n}\left[(\alpha + i\beta n)\kappa_n - \rho_n c v_0\right] = \alpha(\alpha\kappa_0 - Cc) \tag{S25}$$

Then α can be solved numerically from Eq. (S25) with enough accuracy by cutting to the Nth order (we take N = 4 in this work, i.e. m and n both take ±1, ±2, ±3, ±4). With each solution of α known, we then have F_m from Eq. (S23) and obtain the corresponding $f(\chi)$.

Then the temperature solution should be a linear combination of Eq. (S19)

$$T(x,t)=\sum_k C_k e^{\alpha_k x} f_k(\chi)=\sum_{k,m} C_k F_m^k e^{\alpha_k x+im\beta\chi} \tag{S26}$$

where k represents the kth particular solution, and C_k is the corresponding coefficient determined by the boundary conditions at two ends. During the calculation, it can be found that α has two real solutions, which mainly contribute to the profile of temperature distribution. (Other complex solutions can be neglected, for they vanishes as $\beta\to\infty$.)

The case that there exists a net mass flux (C ≠ 0)

Following the above steps, we have the analytical solution of the general 1D convection-diffusion equation. It can be found during the solving process that when $C \neq 0$, α has a nonzero real solution, which indicates a concave/convex temperature profile. Next we perform COMSOL simulations to verify the accuracy of the 4-order analytic solution. We adopt the distributions of ρ and κ as

$$\rho(\chi)=\rho_0\left[1+\Delta_\rho\cos(\beta\chi)\right] \tag{S27}$$

$$\kappa(\chi)=\kappa_0\left[1+\Delta_\kappa\cos(\beta\chi)\right] \tag{S28}$$

The parameters are set as ρ_0 = 2000 kg m^{-3}, c = 1000 J kg^{-1} K^{-1}, κ_0 = 100 W m^{-1} K^{-1} and d = 1 cm. There are two typical values of C, namely 0 and $\rho_0 v_0$ that correspond to cyclic mass movement and uniform motion at constant speed v_0, respectively. Therefore, we calculate solutions for $C/\rho_0 v_0$ = 0, 0.1, 0.2, 0.5, and 1 to cover different levels of average mass flux through the system (Supplementary Figure 1a). The backward (Supplementary Figure 1b) and forward (c) temperature distributions are obtained with Δ_ρ = 0.3, Δ_κ = 0.9, and $\mu = \rho_0 v_0/\kappa_0 = 1/d$. All the lines in Supplementary Figure 1 are analytical solutions, while the scatters are the results of COMSOL simulations. We see that the analytical solutions are in good agreement with the simulation results, which confirms that the 4-order analytical solution is accurate enough. To illustrate the nature of the non-reciprocity for $C \neq 0$, we plot the dependence of α on the modulating speed μ, amplitudes of modulation Δ_κ and Δ_ρ in Supplementary Figure 1d, e, and f, respectively. For $C \neq 0$, it is easy to see that the non-reciprocity is not generated by time modulation, because α is nonzero at $\mu = 0$, $\Delta_\kappa = 0$, and $\Delta_\rho = 0$. On the contrary, α is actually maximized at $\mu = 0$ and $\Delta_\rho = 0$ (Supplementary Figure 1d and f).

The case that no net mass flux exists (C = 0)

When $C = 0$ (the case discussed in the main text), Eq. (S25) becomes

$$\alpha^2\sum_m\left[(\alpha+i\beta m)\kappa_{-m}-\rho_{-m}cv_0\right]\sum_n A_{m,n}\left[(\alpha+i\beta n)\kappa_n-\rho_n cv_0\right]=\alpha^2\kappa_0 \tag{S29}$$

It is easy to find that α has two repeated roots at 0 (and the real solution to α is only 0). So we need to find another particular solution, which is supposed to have the form as

$$T_\#(x,t)=x+f_\#(\chi)=x+\sum_m F_m^\# e^{im\beta\chi} \tag{S30}$$

The details of $f_\#(\chi)$ can be solved by substituting Eq. (S30) into Eq. (S17) (C = 0) and performing similar process as above.

Thus the temperature solution should be

$$T(x,t)=\sum_k C_k e^{\alpha_k x} f_k(\chi)+C_\#\left[x+f_\#(\chi)\right] \tag{S31}$$

where C_k and $C_\#$ are coefficients determined by the boundary conditions.

Recalling the formula to calculate the heat flux

$$q(x,t)=-\kappa(x,t)\frac{\partial T(x,t)}{\partial x}+\left[\rho(x,t)-\rho_0\right]v_0c\left[T(x,t)-T_{\mathrm{ref}}\right] \tag{S32}$$

we have the analytical heat flux

$$\begin{aligned}q(x,t)=&-\left(\sum_n\rho_n e^{in\beta\chi}-\rho_0\right)v_0cT_{\mathrm{ref}}-\sum_{k,n,m}C_k\left[(\alpha_k+i\beta m)\kappa_{n-m}+(\delta_{n,m}-1)\rho_{n-m}v_0c\right]F_m^k e^{\alpha_k x+in\beta\chi}\\&-C_\#\sum_n\left\{\kappa_n+x(\delta_{n.0}-1)\rho_n v_0c+\sum_m\left[im\beta\kappa_{n-m}+(\delta_{n.m}-1)\rho_{n-m}v_0c\right]F_m^\#\right\}e^{in\beta\chi}\end{aligned} \tag{S33}$$

Then we obtain the time-averaged heat flux by letting n = 0, that is

$$\begin{aligned}\langle q(x)\rangle=&-\sum_{k,m}C_k\left[(\alpha_k+i\beta m)\kappa_{-m}+(\delta_{0,m}-1)\rho_{-m}v_0c\right]F_m^k e^{\alpha_k x}\\&-C_\#\left\{\kappa_0+\sum_m\left[im\beta\kappa_{-m}+(\delta_{0.m}-1)\rho_{-m}v_0c\right]F_m^\#\right\}\end{aligned} \tag{S34}$$

Recalling the 0th order of Eq.(S22) (n = 0)

$$\alpha\sum_m\left[(\alpha+i\beta m)\kappa_{-m}+\delta_{0,m}\rho_0v_0c-\rho_{-m}v_0c\right]F_m=0 \tag{S35}$$

It is easy to find that for all $\alpha_k\neq 0$, there exists

$$\sum_m\left[(\alpha_k+i\beta m)\kappa_{-m}+(\delta_{0,m}-1)\rho_{-m}v_0c\right]F_m^k=0 \tag{S36}$$

Besides, considering Eq.(S23), it is easy to find that for α_k = 0, the corresponding F_m^k = 0 ($m\neq 0$), thus Eq.(S36) also holds for α_k = 0. Therefore, the time-averaged heat flux becomes

$$\langle q(x)\rangle = -C_{\#}\left\{\kappa_0 + \sum_m \left[im\beta\kappa_{-m} + \left(\delta_{0.m} - 1\right)\rho_{-m}v_0 c\right] F_m^{\#}\right\} \tag{S37}$$

Noting that $\langle q(x)\rangle$ is a constant independent of x, indicating that the heat flux through the system is a conserved quantity.

The solid lines in Supplementary Figure 2a and b are plotted using Eq. (S33), while the dash lines are plotted using Eq. (S37). For $\mu = 1/d$, the backward and forward time-averaged heat flux $\langle q_\mathrm{b}(x)\rangle$ and $\langle q_\mathrm{f}(x)\rangle$ are calculated as -2.54×10^4 W m^{-2} and 2.54×10^4 W m^{-2}, respectively. For $\mu = 4/d$, $\langle q_\mathrm{b}(x)\rangle$ and $\langle q_\mathrm{f}(x)\rangle$ are -4.26×10^4 W m^{-2} and 4.26×10^4 W m^{-2}, respectively. Clearly, the condition for thermal reciprocity (Eq. (4) in the main text) is satisfied. Therefore, the heat transfer in materials with the first type of density modulation and without net directional flow of mass is reciprocal.

Supplementary Note 3: Analytical solution of Eq. (8)

We also use the temperature distribution in the form as Eq. (S19) to solve the 1D hypothetical diffusion equation[2]

$$\rho(\chi)c\frac{\partial T}{\partial t}=\frac{\partial}{\partial x}\left[\kappa(\chi)\frac{\partial T}{\partial x}\right] \tag{S38}$$

Substituting Eq. (S19) into (S38) gives

$$\kappa f''+\left(2\alpha\kappa+\kappa'+\rho c v_0\right)f'+\left(\alpha\kappa+\kappa'\right)\alpha f=0 \tag{S39}$$

Using the Fourier expansions of the periodic functions ρ, κ and f in Eqs. (S12), (S13) and (S20), we can write the nth order of Eq. (S39) as

$$\sum_m\left[(\alpha+i\beta n)(\alpha+i\beta m)\kappa_{n-m}+i\beta m\rho_{n-m}cv_0\right]F_m=0 \tag{S40}$$

where $n = 0, \pm1, \pm2, \ldots$ Then the solutions to Eq. (S39) is equivalent to that of the matrix equation as following

$$\left[G_{n,m}\right]\left[F_m\right]=[0] \tag{S41}$$

where $[G_{n,m}]$ is defined as

$$G_{n,m}=(\alpha+i\beta n)(\alpha+i\beta m)\kappa_{n-m}+i\beta m\rho_{n-m}cv_0 \tag{S42}$$

By numerically solving α and F_m up to the fourth order (n, $m = \pm1, \pm2, \pm3, \pm4$) from Eq. (S41), we can obtain the temperature solution (the same form as Eq. (S26)). The results are in good agreement with numerical simulations as shown in the main text.

For the conductive heat flux is the only constituent of energy flux in the system, we have the total heat flux $q(x,t)$ as following based on the analytical temperature solution

$$q(x,t)=-\sum_{k,n,m}C_k\left(\alpha_k+i\beta m\right)\kappa_{n-m}F_m^k e^{\alpha_k x+in\beta\chi} \tag{S43}$$

Clearly, the average of heat flux over time is the 0th order of Eq. (S43), that is

$$\langle q(x)\rangle=-\sum_{k,m}C_k\left(\alpha_k+i\beta m\right)\kappa_{-m}F_m^k e^{\alpha_k x} \tag{S44}$$

The solid lines in Supplementary Figure 2c and d are plotted using Eq. (S43), while the dash lines are plotted using Eq. (S44). It is noted that at the two ends of the system, the average heat fluxes $\langle q(0)\rangle$ and $\langle q(L)\rangle$ are obviously not equal. The difference clearly shows that additional energy input or extraction is required to compensate it, which is also hard to implement in practice.

Supplementary Note 4: Analytical model and solution of Eq. (9)

For the second type of modulation, we consider mass motion along y with speed $v_y(x,y,t)$ to modulate the density locally. The 2D convection-diffusion satisfies

$$\rho(\zeta)c\frac{\partial T}{\partial t}+\rho(\zeta)cv_y\frac{\partial T}{\partial y}=\frac{\partial}{\partial x}\left[\kappa^x(\zeta)\frac{\partial T}{\partial x}\right]+\frac{\partial}{\partial y}\left[\kappa^y(\zeta)\frac{\partial T}{\partial y}\right] \quad \text{(S45)}$$

where $\zeta = x + \eta y - v_0 t$. According to the continuity equation $\partial\rho(\zeta)/\partial t + \partial[\rho(\zeta)v_y]/\partial y = 0$, we find $\rho v_y = (\rho - \rho_0)v_{0y} + C$, where $v_{0y} = v_0/\eta$. Substituting it into Eq. (S45), we have the general 2D convection-diffusion equation

$$\rho(\zeta)c\frac{\partial T}{\partial t}+\left\{\left[\rho(\zeta)-\rho_0\right]v_{0y}+C\right\}c\frac{\partial T}{\partial y}=\frac{\partial}{\partial x}\left[\kappa^x(\zeta)\frac{\partial T}{\partial x}\right]+\frac{\partial}{\partial y}\left[\kappa^y(\zeta)\frac{\partial T}{\partial y}\right] \quad \text{(S46)}$$

Consider the general form of the solution

$$T(x,y,t)=e^{\alpha x}f(\zeta) \quad \text{(S47)}$$

Substituting Eq. (S47) into (S46) gives

$$\left(\kappa^x+\eta^2\kappa^y\right)f''+\left[2\alpha\kappa^x+\kappa^{x\prime}+\eta^2\kappa^{y\prime}+\left(\rho_0 v_0-\eta C\right)c\right]f'+\left(\alpha\kappa^x+\kappa^{x\prime}\right)\alpha f=0 \quad \text{(S48)}$$

The nth order of its Fourier expansion is

$$\sum_m\left[\left(\alpha+i\beta n\right)\left(\alpha+i\beta m\right)\kappa^x_{n-m}-\eta^2\beta^2 nm\kappa^y_{n-m}+\delta_{n,m}i\beta n\left(\rho_0 v_0-\eta C\right)c\right]F_m=0 \quad \text{(S49)}$$

For $n \neq 0$ and $m \neq 0$, F_m can be solved as

$$F_m=-\sum_{n\neq 0}B_{n,m}\left(\alpha+i\beta n\right)\alpha\kappa^x_n F_0 \quad \text{(S50)}$$

where $B_{m,n}$ is the m-row, n-column element of a matrix $[B_{m,n}]$, which is defined as

$$\left[B_{n,m}\right]=\left[\left(\alpha+i\beta n\right)\left(\alpha+i\beta m\right)\kappa^x_{n-m}-\eta^2\beta^2 nm\kappa^y_{n-m}+\delta_{n,m}i\beta n\left(\rho_0 v_0-\eta C\right)c\right]^{-1} \quad \text{(S51)}$$

Substituting Eq. (S50) into the 0th order of Eq. (S49) (n = 0) gives

$$\alpha^2\sum_{m\neq 0}\left(\alpha+i\beta m\right)\kappa^x_{-m}\sum_{n\neq 0}B_{n,m}\left(\alpha+i\beta n\right)\kappa^x_n=\alpha^2\kappa^x_0 \quad \text{(S52)}$$

It is easy to find that α has two repeated roots at zero. So we need to find another particular solution, which is supposed to have the form as

$$T_\#(x,y,t)=x+f_\#(\zeta) \quad \text{(S53)}$$

Thus the temperature solution should be (similar to the 1D case)

$$T(x,y,t)=\sum_k C_k e^{\alpha_k x}f_k(\zeta)+C_\#\left[x+f_\#(\zeta)\right] \quad \text{(S54)}$$

If ρ and κ are specified and boundary conditions are given, it can be solved to get more detailed results with the same technique as above. For $\alpha = 0$ is the only real solution and independent of C, it implies that the general 2D convection-diffusion system is reciprocal, including the case in the main text ($C = \rho_0 v_{0y}$). In addition, with the property of Eq.(S54): $v_{0y}\partial T/\partial y = -\partial T/\partial t$, Eq. (S46) can be transformed to

$$\frac{\partial}{\partial x}\left[\kappa^x(\zeta)\frac{\partial T}{\partial x}\right]+\frac{\partial}{\partial y}\left[\kappa^y(\zeta)\frac{\partial T}{\partial y}\right]=0 \tag{S55}$$

Since the density disappears in Eq. (S55), the modulation of thermal conductivity alone is insufficient to generate thermal non-reciprocity. This analysis also clearly demonstrates how this class of density modulation cannot break reciprocity.

The parameters for the results in Fig. 3c and d (lines) of the main text are set as Eq. (S60)-(S62). Note that many orders of the Fourier series are required to accurately describe a square wave, so we directly performed numerical simulations of the 2D model instead of calculating the analytical solutions.

Supplementary Note 5: Analytical model of the 3D implementation

For convenience, we assume that the 3D setup (density ρ_A = 8390 kg m^{-3}, heat capacity c_A = 375 J kg^{-1} K^{-1}, and thermal conductivity κ_A = 123 W m^{-1} K^{-1}) is put in air (density ρ_B = 1.3 kg m^{-3}, heat capacity c_B = 1016 J kg^{-1} K^{-1}, and thermal conductivity κ_B = 0.025 W m^{-1} K^{-1}). By projecting the side surface $r = R_2$ of the 3D model onto a plane (x, y), we obtain the 2D thermal conductivity distribution on a slice $2n\delta \leq x \leq 2(n + 1)\delta$, $-\pi R_2/4 \leq y \leq \pi R_2/4$ containing a fixed plate and its adjacent region, where n = 0, 1, … The product of density and heat capacity and the thermal conductivity of the fixed plate are always

$$\rho c_{\text{fix}}\left(x,y,t\right)=\rho_A c_A \tag{S56}$$

$$\kappa_{\text{fix}}\left(x,y,t\right)=\kappa_A \tag{S57}$$

For the adjacent region where the moving plates could enter and leave, the material parameters are square wave distributions

$$\rho c_{\text{mov}}\left(x,y,t\right)=\rho_B c_B+\left(\rho_A c_A-\rho_B c_B\right)\text{rect}\left(\zeta\right) \tag{S58}$$

$$\kappa_{\text{mov}}\left(x,y,t\right)=\kappa_B+\left(\kappa_A-\kappa_B\right)\text{rect}\left(\zeta\right) \tag{S59}$$

where rect(ζ) is a square wave with wavelength d, $\zeta = x + \eta y - v_0 t$, $\eta = d/(\pi R_2)$, and $v_0 = \Omega R_2 \eta$. To simplify the distributions, we propose that the effective material parameters of two adjacent slices can be estimated as

$$\rho c\left(\zeta\right)=\left(\rho c_{\text{fix}}+\rho c_{\text{mov}}\right)/2 \tag{S60}$$

$$\kappa^x\left(\zeta\right)=2\left(\kappa_{\text{fix}}^{-1}+\kappa_{\text{mov}}^{-1}\right)^{-1} \tag{S61}$$

$$\kappa^y\left(\zeta\right)=\left(\kappa_{\text{fix}}+\kappa_{\text{mov}}\right)/2 \tag{S62}$$

Assuming $d/\delta \rightarrow \infty$, the governing equation should be written as

$$\rho c\left(\zeta\right)\frac{\partial T}{\partial t}+\frac{1}{2}\rho c_{\text{mov}}\left(\zeta\right)v_{0y}\frac{\partial T}{\partial y}=\frac{\partial}{\partial x}\left[\kappa^x\left(\zeta\right)\frac{\partial T}{\partial x}\right]+\frac{\partial}{\partial y}\left[\kappa^y\left(\zeta\right)\frac{\partial T}{\partial y}\right] \tag{S63}$$

where $v_{0y} = \Omega R_2$. We can also prove the thermal reciprocity of this model by analyzing the time-averaged heat fluxes along x direction in forward and backward regimes, i.e., $\langle q_f \rangle + \langle q_b \rangle = -\kappa^x \partial(T_f + T_b)/\partial x = -\kappa^x \partial(T_{hot} + T_{cold})/\partial x = 0$, as confirmed in Supplementary Figure 3a.

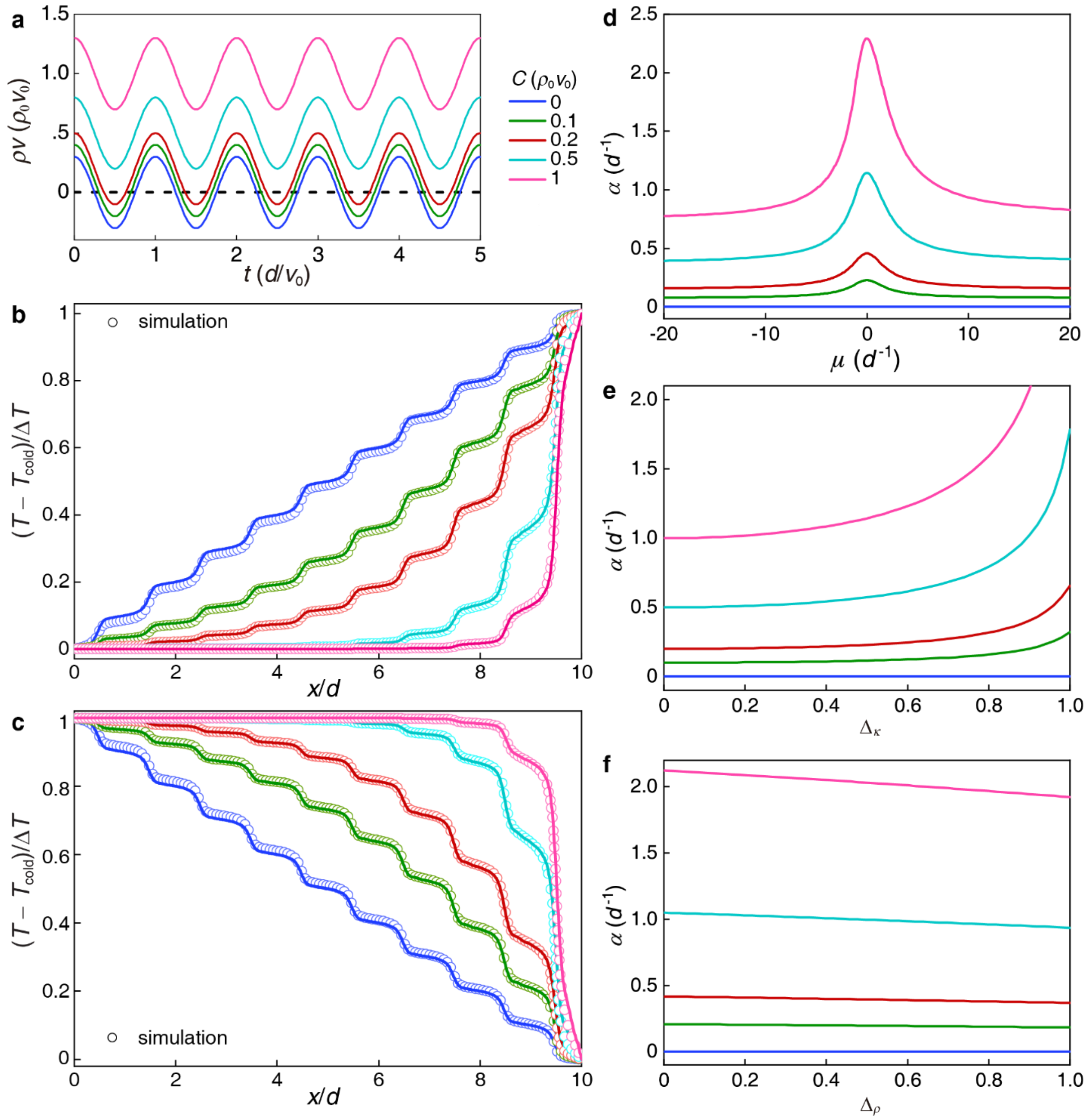

Supplementary Figure 1 Heat transfer under 1D density modulation with nonzero average mass flux. a For $C \neq 0$, there is an additional mass flux through the system. **b-c** Backward (**b**) and forward (**c**) temperature distributions. The scatters are results of numerical simulations. **d-f** The asymmetry of temperature distributions (characterized by α) for different modulation speed μ (**d**), and amplitudes of modulation Δ_κ (**e**) and density Δ_ρ (**f**).

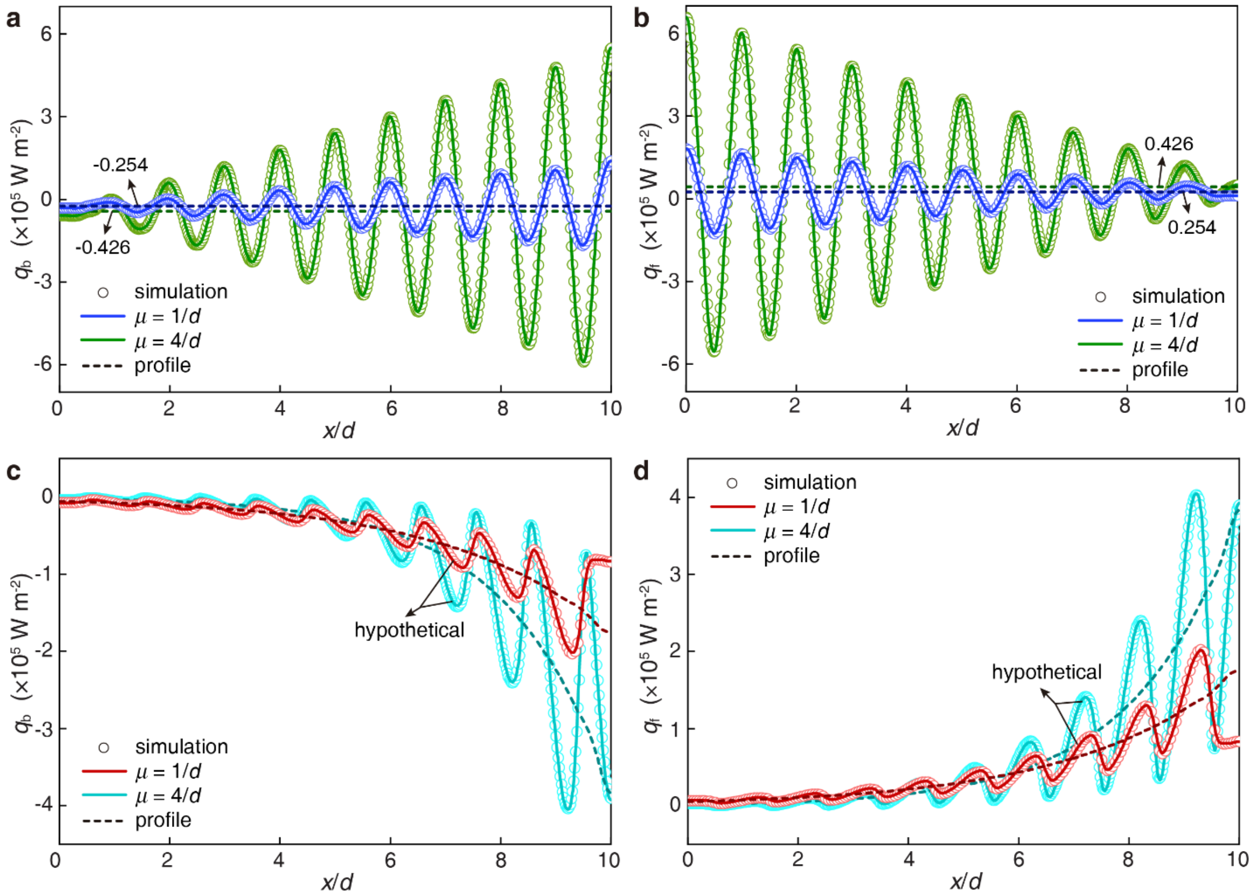

Supplementary Figure 2 Heat flux under 1D density modulation. a-b Backward (**a**) and forward (**b**) heat flux distributions of the system described by Eq. (5). The time-averaged heat flux is a constant quantity independent of x. For $\mu = 1/d$, the analytical value of time-averaged heat flux density is $\langle q_b \rangle = -2.54 \times 10^4$ W m^{-2} (backward) and $\langle q_f \rangle = 2.54 \times 10^4$ W m^{-2} (forward), while for $\mu = 4/d$, $\langle q_b \rangle = -4.26 \times 10^4$ W m^{-2} (backward) and $\langle q_f \rangle = 4.26 \times 10^4$ W m^{-2} (forward). It is easy to find that $\langle q_b \rangle = -\langle q_f \rangle$, satisfying Eq. (4) in the main text and demonstrating reciprocity in heat transfer. **c-d** Backward (**c**) and forward (**d**) heat flux distributions of the virtual system described by Eq. (8), discussed in an insightful work[2] that indicates thermal non-reciprocity at a mathematical level. Although the heat flux distributions show nonreciprocal heat transfer, however, it is hypothetical because density modulation cannot be achieved at no cost, and the implementation of modulation will inevitably change the governing equation. The heat flux is obviously not a conserved quantity under this circumstance since its time-averaged profile is not constant, implying that additional energy input or extraction is required to compensate it. The results are plotted at $t = Nd/v_0$ (N is a large enough integer to achieve time-harmonic steady state).

Scatter points are simulated results, lines are analytical results, and dashed lines are analytical solutions of time-averaged heat flux.

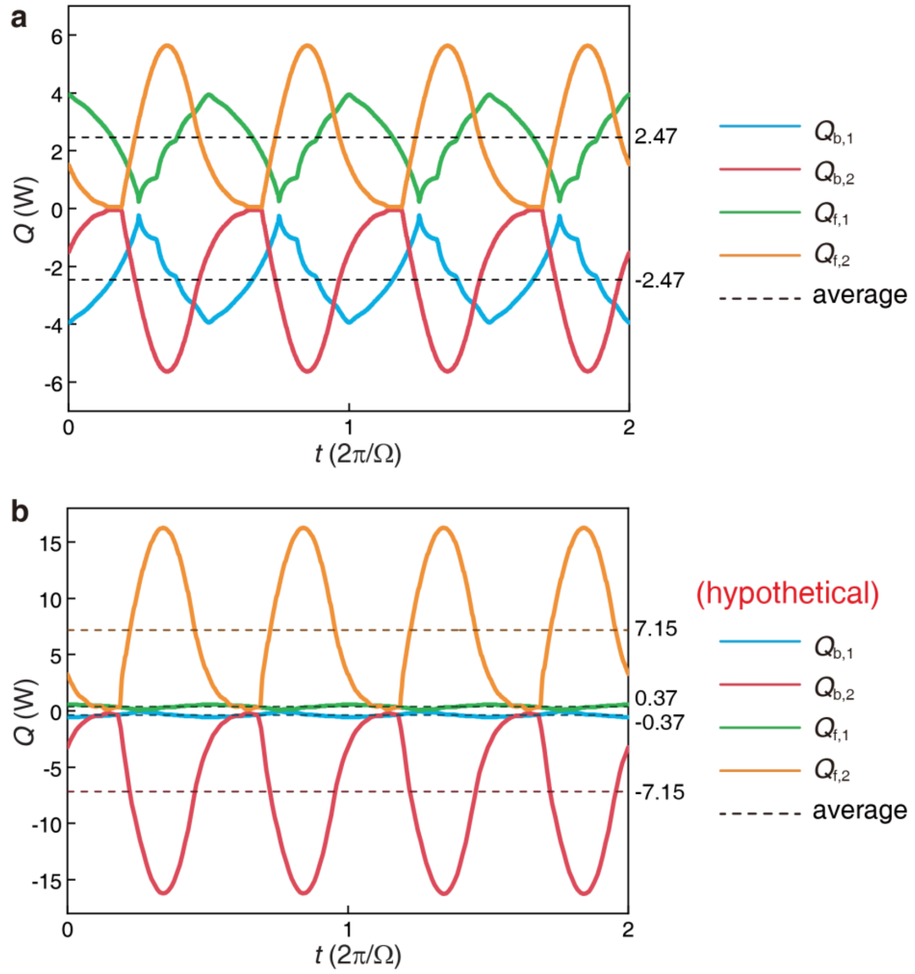

Supplementary Figure 3 Heat flow under 3D density modulation. a Backward and forward heat flow (Q_b and Q_f) at two ends (represented by 1 and 2) of the system. The average heat flow into one port of the system equals to that out of the other port. According to the numerical results, the average heat flow is $\langle Q_{b,1}\rangle = \langle Q_{b,2}\rangle = -2.47$ W and $\langle Q_{f,1}\rangle = \langle Q_{f,2}\rangle = 2.47$ W, which meets the condition for thermal reciprocity (Eq. (4) in the main text). **b** Backward and forward heat flow at both ends of the hypothetical system where the moving plates have time-varying masses, violating the law of mass conservation. Numerical results give that $\langle Q_{b,1}\rangle = -0.37$ W, $\langle Q_{b,2}\rangle = -7.15$ W, $\langle Q_{f,1}\rangle = 0.37$ W, $\langle Q_{f,2}\rangle = 7.15$ W. The figures are plotted with numerical results by COMSOL simulation after achieving the time-harmonic steady state. The heat flow is obtained by calculating a surface integral over the fan-shaped region at the ends of the system. and subscripted 1 and 2 represent the position $x = 0$ and $x = L$, respectively.

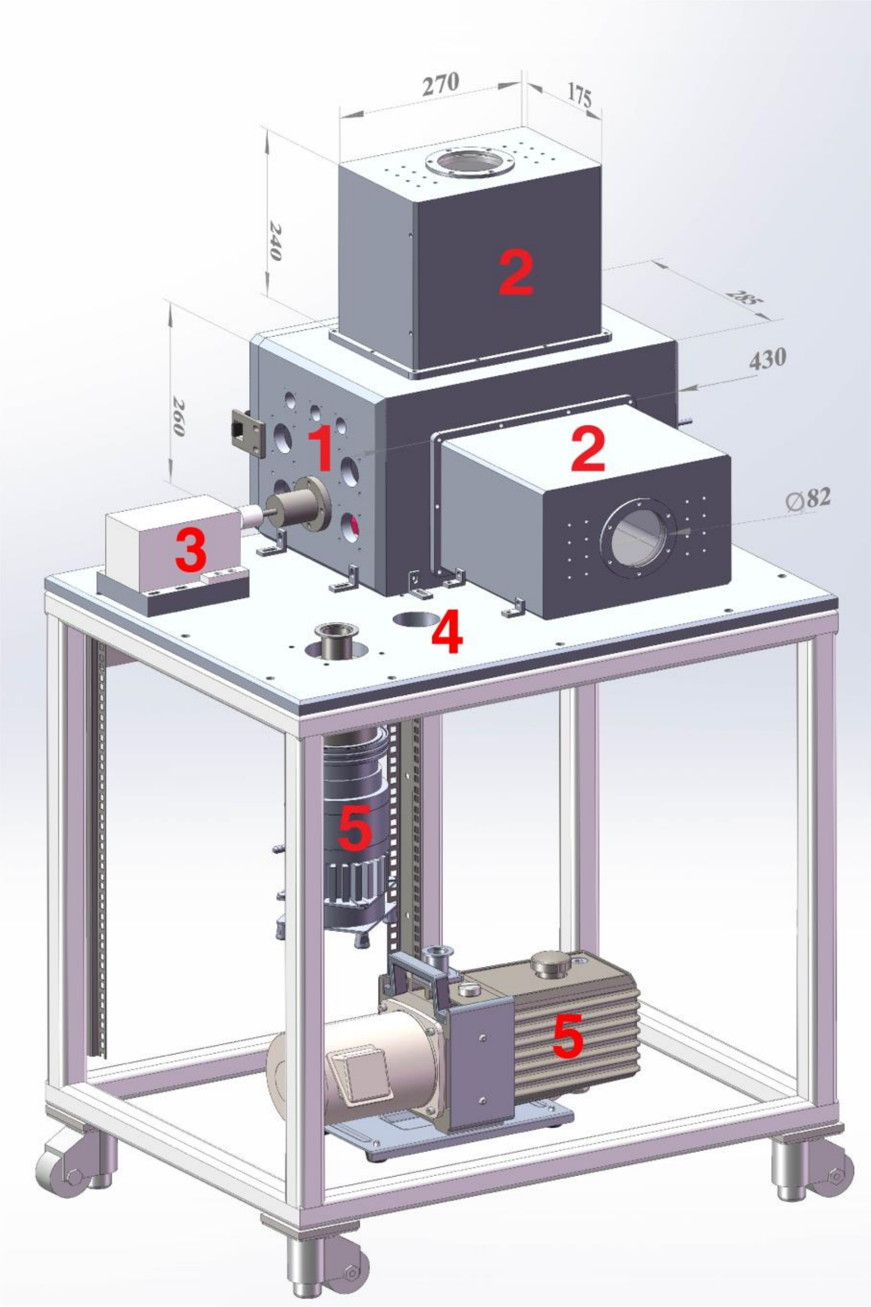

Supplementary Figure 4 Structural design of the vacuum chamber. 1. The experimental system is set inside the vacuum chamber (430×285×260 mm^3), and the lining of the vacuum chamber is coated to reduce the reflectivity. **2**. Two observation windows are fabricated to measure the temperature profiles of the whole system. The size of arms is 270×175×240 mm^3 and the diameter of the circular germanium glass windows is 82 mm. **3**. The rotator driving the system is set outside the vacuum chamber for better heat dissipation. **4**. Supporting platform. **5**. Molecular pump LF-110 and mechanical pump BSV-16 are employed to reduce the density of air in the vacuum chamber as low as 10^{-3} Pa.

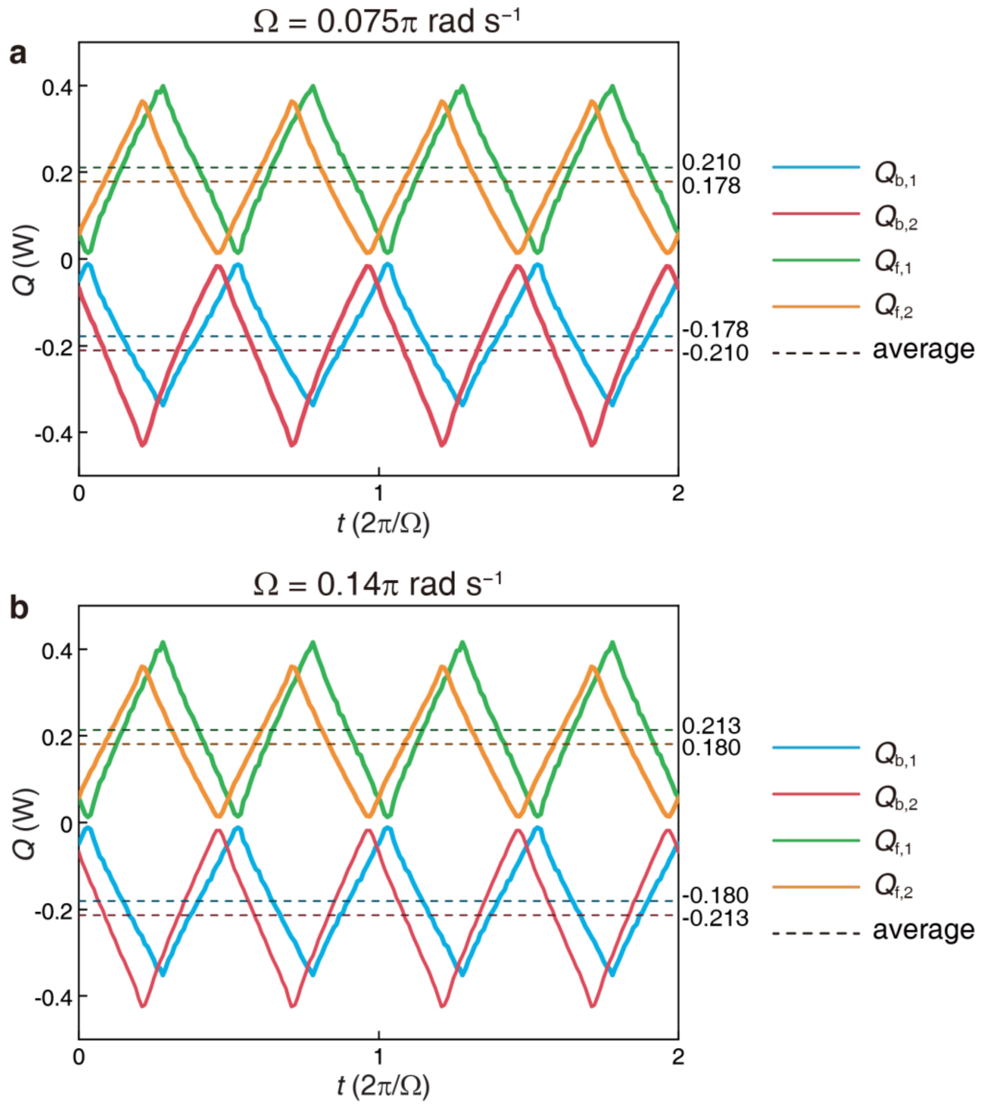

Supplementary Figure 5 Verification of reciprocal heat flow in the experiment. a-b Backward and forward heat flow (Q_b and Q_f) at two ports (represented by 1 and 2) for angular velocity Ω = 0.075π rad s^{-1} (**a**) and 0.14π rad s^{-1} (**b**). According to the numerical results, for Ω = 0.075π rad s^{-1}, the average heat flow is $\langle Q_{b,1}\rangle = -0.178$ W, $\langle Q_{b,2}\rangle = -0.210$ W, $\langle Q_{f,1}\rangle = 0.210$ W, $\langle Q_{f,2}\rangle$ = 0.178 W. For Ω = 0.14π rad s^{-1}, the average heat flow is $\langle Q_{b,1}\rangle = -0.180$ W, $\langle Q_{b,2}\rangle = -0.213$ W, $\langle Q_{f,1}\rangle = 0.213$ W, $\langle Q_{f,2}\rangle = 0.180$ W. It is easy to check that $\langle Q_{b,1}\rangle = -\langle Q_{f,2}\rangle$ and $\langle Q_{b,2}\rangle = -\langle Q_{f,1}\rangle$, which meets the condition for thermal reciprocity (Eq. (4) in the main text). The figures are plotted with numerical results by COMSOL simulation using the same parameter settings as in the experiment. The heat flow is obtained by calculating a surface integral over the fan-shaped region in contact with the two heat sources, and subscripted 1 and 2 represent the left and right ends, respectively.

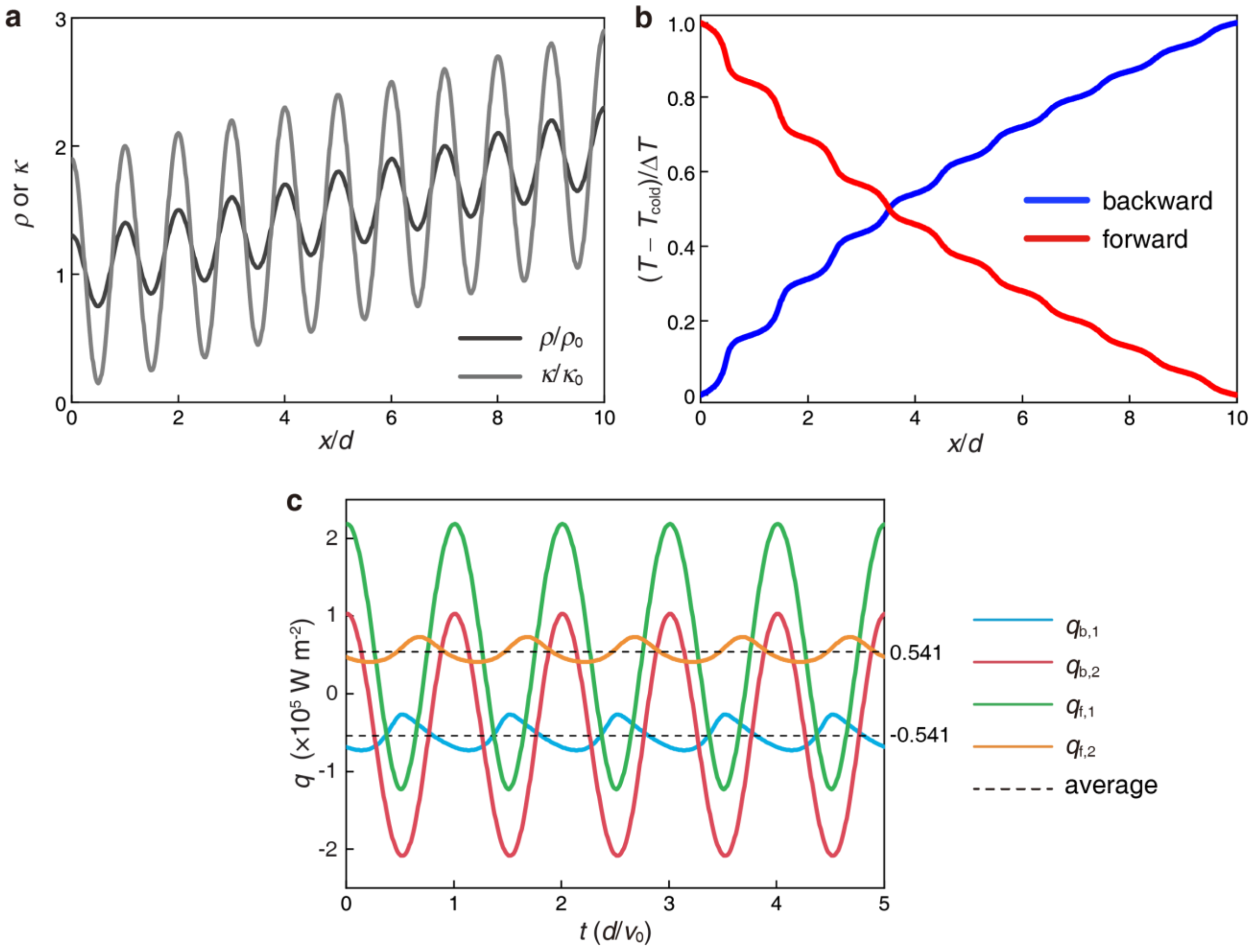

Supplementary Figure 6 Heat transfer under time modulation of a parity asymmetric system. **a** Density ρ and thermal conductivity κ distributions of the system at $t = Nd/v_0$ (N is an integer). **b** Backward and forward temperature distributions of the system. **c** Backward and forward heat flux at both ends of the system. The average heat fluxes at two ports 1 and 2 are equal: $\langle q_{b,1} \rangle = \langle q_{b,2} \rangle = -5.41 \times 10^4$ W m^{-2} (backward) and $\langle q_{f,1} \rangle = \langle q_{f,2} \rangle = 5.41 \times 10^4$ W m^{-2} (forward). Although the temperature distributions are asymmetric, this system is thermal reciprocal, for the forward and backward average heat fluxes meet the condition for thermal reciprocity (Eq. (4) in the main text). The results are numerical results by COMSOL simulation after achieving the time-harmonic steady state. The parameters are set as follows: $\rho(x,t) = \rho_0[x/L + 1 + \Delta_\rho \cos(\beta\chi)]$ and $\kappa(x,t) = \kappa_0[x/L + 1 + \Delta_\kappa \cos(\beta\chi)]$, where $\beta = 2\pi/d$, $\rho_0 = 2000$ kg m^{-3}, $\Delta_\rho = 0.3$, $\kappa_0 = 100$ W m^{-1} K^{-1}, $\Delta_\kappa = 0.9$. $d = 1$ cm and $L = 10d$. The specific heat capacity is $c = 1000$ J kg^{-1} K^{-1}. The modulation speed is $v_0 = \mu\kappa_0/\rho_0 c$ with $\mu = 1/d$. Constant temperatures at two boundaries are set as $T_{cold} = 273$ K and $T_{hot} = 323$ K. 1 and 2 represent the position $x = 0$ and $x = L$, respectively.

Supplementary References

1. Bejan, A, Convection Heat Transfer (John wiley & sons, 2013).
2. Torrent, D., Poncelet, O. & Batsale, J.-C. Nonreciprocal Thermal Material by Spatiotemporal Modulation. Phys. Rev. Lett. 120, 125501 (2018).
