# Supplementary material for: Reciprocity of thermal diffusion in time-modulated systems
Source: Nat Commun. 2022 Jan 10;13:167. doi: 10.1038/s41467-021-27903-3 (PMC8748696; doi:10.1038/s41467-021-27903-3)
Supplement: Supplementary file 3 — Description of Additional Supplementary Files [file 41467_2021_27903_MOESM3_ESM.docx]

Description of Additional Supplementary Files

Title: Supplementary Movie 1:

Description: Evolution of the temperature distributions on the system surface (upper) and along the top line (lower) for backward (left)and forward (right) heat transfer for the physical 3D model.

Title: Supplementary Movie 2:

Description: Evolution of the temperature distributions on the system surface (upper) and along the top line (lower) for backward (left)and forward (right) heat transfer for the unphysical 3D model where the mass of the moving plates are artificially varied with time.

Title: Supplementary Movie 3:

Description: The experimental system in movement (top), with the temperature distributions on it for backward (middle) and forward (bottem) heat transfer.
